# Impact of Lymph Node Dissection on Postoperative Complications of Total Thyroidectomy in Patients with Thyroid Carcinoma

**DOI:** 10.3390/cancers14215462

**Published:** 2022-11-07

**Authors:** Gregory Baud, Arnaud Jannin, Camille Marciniak, Benjamin Chevalier, Christine Do Cao, Emmanuelle Leteurtre, Amandine Beron, Georges Lion, Samuel Boury, Sebastien Aubert, Brigitte Bouchindhomme, Marie-Christine Vantyghem, Robert Caiazzo, François Pattou

**Affiliations:** 1 Department of Endocrine Surgery, CHU Lille, F-59000 Lille, France; 2 Department of Endocrinology and Metabolism, CHU Lille, F-59000 Lille, France; 3 Pathology Institute, Biology Pathology Center, CHU Lille, F-59000 Lille, France; 4 Department of Nuclear Medicine, CHU Lille, F-59000 Lille, France; 5 Department of Radiology, CHU Lille, F-59000 Lille, France

**Keywords:** thyroid carcinoma, lymph node dissection, laryngeal nerve injury, hypoparathyroidism

## Abstract

**Simple Summary:**

Thyroid cancers are the most common endocrine cancers and their incidence has increased significantly in recent decades worldwide. Total thyroidectomy is recognized as the gold standard treatment for thyroid cancers. However, controversies remain regarding lymph node dissection and the related morbidities. The aim of our retrospective study was to analyse the risk factors of post-thyroidectomy complications and to assess the morbidity of lymph node dissection, especially in the central neck compartment, since prophylactic central lymph node dissection has not been proven to bring an overall survival benefit. In a cohort of 1547 patients with thyroid carcinoma who underwent total thyroidectomy with or without lymph node dissection, we found that lymph node dissection in the central and lateral compartments, respectively, increased the risk of transient and permanent hypoparathyroidism and recurrent laryngeal nerves injuries.

**Abstract:**

Background: Lymph node dissection (LND) in primary treatment of differentiated thyroid carcinoma is controversial. The aim of our retrospective study was to analyse the risk factors of post-thyroidectomy complications and to assess the morbidity of lymph node dissection, especially in the central neck compartment, since prophylactic central lymph node dissection has not been proven to bring an overall survival benefit. Methods: We performed a retrospective analysis of postoperative complications from 1547 consecutive patients with differentiated thyroid carcinoma in an academic department of endocrine surgery over a period of 10 years. Results: A total of 535 patients underwent lymph node dissection, whereas the other 1012 did not. The rate of postoperative hypoparathyroidism was higher in patients with LND (17.6% vs. 11.4%, *p* = 0.001). No significant difference in the rate of permanent hypoparathyroidism (2.4% vs. 1.3%, *p* = 0.096) was observed between these two groups. A multivariate analysis was performed. Female gender, ipsilateral and bilateral central LND (CLND), parathyroid autotransplantation, and the presence of the parathyroid gland on the resected thyroid were associated with transient hypoparathyroidism. Bilateral CLND and the presence of the parathyroid gland on specimen were associated with permanent hypoparathyroidism. The rate of transient recurrent laryngeal nerve (RLN) injury (15.3% vs. 5.4%, *p* < 0.001) and permanent RLN injury (6.5% vs. 0.9%, *p* < 0.001) were higher in the LND group. In multivariate analysis, ipsilateral and bilateral lateral LND (LLND) were the main predictive factors of transient and permanent RLN injury. Bilateral RLN injury (2.6% vs. 0.4%, *p* < 0.001), chyle leakage (2.4% vs. 0%, *p* < 0.001), other nerve injuries (2.2% vs. 0%, *p* < 0.001), and abscess (2.4% vs. 0.5%, *p* = 0.001) were higher in the patients with LND. Conclusions: The surgical technique and the extent of lymph node dissection during surgery for thyroid carcinoma increase postoperative morbidity. A wider knowledge of lymph-node-dissection-related complications associated with thyroid surgery could help surgeons to carefully evaluate the surgical and medical therapeutic options.

## 1. Introduction

Thyroid carcinoma is the most common endocrine cancer and its incidence has dramatically increased in recent decades all over the world [1]. Nowadays, total thyroidectomy (TT) has been accepted as the most efficient procedure for treating thyroid cancer. However, controversy remains regarding the optimal extent of thyroid surgery and lymph node dissection. TT with therapeutic lymph node dissection (LND) is recommended in patients with lymph node metastases in the neck identified on preoperative examination or at the time of surgery [2]. Nevertheless, the role of prophylactic LND in patients with N0 neck is still uncertain [3,4,5]. Indeed, TT with LND could decrease locoregional recurrence in patients with thyroid carcinoma [6,7,8,9] and could have some important prognostic values for accurate clinical staging and postoperative treatment [10]. However, LND does not seem to affect long-term survival [11,12]. Conversely, TT with LND is associated with a high incidence of transient or permanent hypoparathyroidism and increased risk of injury to the inferior recurrent laryngeal nerve, leading to transient or permanent vocal cord palsy [7,8,9]. Therefore, the aim of this study was to precisely characterise the morbidity of LND and to analyse the predictive risk factors of those relevant complications.

## 2. Patients and Methods

In this retrospective cohort study, we enrolled all consecutive patients with thyroid carcinoma who underwent total thyroidectomy in the Department of General and Endocrine Surgery of the University Hospital of Lille between 1 January 2000 and 31 December 2009. This research project was added to the register of data analysis of the University Hospital of Lille under the reference DEC22-226. The inclusion criteria combined (1) a diagnosis of thyroid carcinoma confirmed by pathology, whether or not malignancy was suspected prior to surgery by preoperative ultrasound and/or preoperative cytology, and (2) a total thyroidectomy, with or without LND in the central and/or lateral neck compartments. Patients were excluded when (1) a unilateral thyroidectomy was performed, (2) they had previous history of thyroid and/or parathyroid surgery, or (3) they had a associated diagnosis of hyperparathyroidism.

### 2.1. Operative Technique

All operations were performed by surgeons with different levels of experience and consisted of a total thyroidectomy after a cervical Kocher incision; the infrahyoid muscles were opened along the midline and muscles were divided as necessary. In all patients, every effort was made to identify and preserve the recurrent laryngeal nerves and the four parathyroid glands. When vascularization of a parathyroid gland appeared compromised, it was selectively resected and autotransplanted in the ipsilateral sternocleidomastoid muscle as previously described [13]. The extent of central neck and lateral neck dissection was determined in accordance with the American Thyroid Association thyroid cancer guidelines at the time of surgery [2]. Prophylactic lymph node dissection for the central neck compartment was performed in patients for suspect malignant thyroid tumor with clinically node-negative disease, primarily in the ipsilateral compartment as previously described [14]. Therapeutic central neck dissection (CND) was performed when abnormal lymphadenopathy was detected during the preoperative or intraoperative examination. In patients with suspected malignant thyroid tumor, lymph node sampling (LNS) for frozen section could be performed in the ipsilateral central or lateral compartments [15]. Lateral neck dissection was performed only in patients with cytology-proven metastatic lymph nodes in the lateral neck, including levels II–V.

### 2.2. Follow-Up

Postoperative follow-up was previously described in Pattou et al. [13]. Briefly, vocal fold function was assessed in all patients by laryngoscopy examination before and immediately after surgery. Recurrent laryngeal nerve injuries were defined as a dysfunction or a total absence of vocal cord mobility compared to the contralateral one based on preoperative fiberoptic laryngoscopy. All vocal fold palsies lasting for more than one year were considered as permanent. The fasting serum calcium and serum phosphorus levels were measured daily before and after surgery until the day of hospital discharge. Hypoparathyroidism was defined as a serum calcium level under 8.0 mg/dL (2 mmol/L) on at least two consecutive measurements and was systematically treated with calcium supplementation. Permanent hypoparathyroidism was defined by the requirement for vitamin D or calcium supplementation (or both) to maintain eucalcemia 1 year after thyroidectomy. All patients were also systematically examined postoperatively for other relevant complications such as hematoma, chyle leakage and nerve damage (spinal accessory nerve, vagus nerve, phrenic nerve, and/or sympathetic trunk).

### 2.3. Statistical Analysis

Continuous variables were expressed as means with standard deviations, and compared with Student’s *t*-test. Nominal data were compared using a Chi-squared test or Fisher’s exact test, as appropriate. Predictive factors of surgical complications with a *p* value < 0.2 in univariate analysis were considered for multivariate model using a downward stepwise binary logistic regression analysis. Differences were considered significant for *p* values inferior to 0.05. All statistical analysis was conducted with SPSS v20 software (SPSS, Chicago, IL, USA).

## 3. Results

### 3.1. Patient Characteristics

The characteristics of the 1547 patients with thyroid carcinoma confirmed by histological analysis of the surgical specimen who underwent total thyroidectomy with or without LND are summarized in Table 1. Among them, 535 (34.6%) patients had LND. LND in the central compartment consisted of 39 (2.5%) ipsilateral central lymph nodes sampling (CLNS), 147 (9.5%) ipsilateral CLND, and 194 (12.5%) bilateral CLND. LND in the lateral compartment consisted of 64 (4.1%) ipsilateral lateral lymph nodes sampling (LLNS), 124 (8.0%) bilateral LLNS, 137 (8.9%) ipsilateral LLND and 57 (3.7%) bilateral LLND. The surgery-related postoperative morbidity is listed in Table 2.

### 3.2. Postoperative Hypoparathyroidism

Transient or permanent hypoparathyroidism occurred, respectively, in 209 (13.5%) and 26 (1.7%) patients (Table 2). The rate of transient hypoparathyroidism was higher in patients with LND in comparison with those without LND (*p* < 0.001) (Table 2). Univariate analysis of predictive factors associated with transient hypoparathyroidism is presented in Table 3. Briefly, we observed that patients who presented with transient postoperative hypoparathyroidism underwent more ipsi (*p* = 0.003) or bilateral (*p* < 0.001) CLND and ipsilateral LLND (*p* = 0.001). Noteworthily, parathyroid glands were more frequently autotransplanted (*p* < 0.001) or present on the surgical specimen (*p* < 0.001) in patients with transient hypoparathyroidism, while the number of parathyroid glands seen during the intervention were similar between the two study groups (*p* = 0.064). Because most of the patients who were submitted to LLND had an ipsi or bilateral CLND, we performed a multivariate analysis to precisely determine the role of each type of LND and other cofactors on the onset of transient postoperative hypoparathyroidism. We found that female gender (*p* < 0.001), ipsilateral CLND (*p* = 0.025), bilateral CLND (*p* < 0.003), parathyroid autotransplantation (*p* = 0.007) and the presence of parathyroid glands on the resected thyroid (*p* < 0.001) were independent predictive factors associated with transient hypoparathyroidism (Table 4). By including all types of lymph node dissection (i.e., lymph node sampling plus lymph node dissection), no significant difference in the rate of permanent hypoparathyroidism was observed between the two groups (*p* = 0.096) (Table 2). However, univariate analysis of predictive factors associated with permanent hypoparathyroidism revealed that patients with permanent postoperative hypoparathyroidism had more bilateral CLND than those with normal parathyroid function (*p* = 0.001) (Table 3). Parathyroid glands were also more frequently retrieved after surgical specimen examination in patients with permanent hypoparathyroidism (*p* < 0.001). Multivariate analysis confirmed that bilateral CLND (*p* = 0.033) and the presence of the parathyroid gland on the resected thyroid (*p* = 0.002) were the two major independent predictive factors of permanent postoperative hypoparathyroidism (Table 4).

### 3.3. Postoperative Recurrent Laryngeal Nerve Injuries

Among all patients studied, transient, permanent or bilateral RLN injuries occurred, respectively, in 137 (8.9%), 44 (2.8%) and 18 (1.1%) patients (Table 2). Patients submitted to total thyroidectomy with LND presented with higher rates of transient (15.3% vs. 5.4%, *p* < 0.001), permanent (6.5% vs. 0.9%, *p* < 0.001) or bilateral (2.6% vs. 0.4%, *p* < 0.001) RLN injuries than those operated on thyroidectomy alone (Table 2). Univariate analysis of predictive factors associated with transient, permanent or bilateral RLN injuries are presented in Table 5. In summary, we observed that ipsilateral and bilateral CLND both increased the rate of transient (*p* < 0.001 and *p* < 0.001, respectively) or permanent RLN injuries (*p* = 0.008 and *p* < 0.001, respectively) (Table 5), whereas only bilateral CLND increased the rate of bilateral RLN injuries (*p* < 0.001) (Table 5). Concerning lateral lymph node dissection, we found that ipsilateral and bilateral LLND increased the rate of transient (*p* < 0.001 and *p* < 0.001, respectively) or permanent RLN injuries (*p* < 0.001 and *p* < 0.001, respectively) (Table 5). Noteworthily, bilateral LLNS also lead to more transient RLN injuries (*p* = 0.020) (Table 5). As for central lymph node dissection, only bilateral LLND increased the rate of bilateral RLN injuries (*p* < 0.001) (Table 5). In multivariate analysis, we demonstrated that bilateral LLND, which exposes both vagus nerves, was the major independent predictive factor of transient (*p* < 0.001), permanent (O *p* < 0.001) or bilateral (*p* < 0.001) RLN injuries (Table 6). In patients who presented with bilateral RLN injuries, initial intervention included thyroidectomy with CLND (*n* = 13) and/or ipsilateral (*n* = 3) or bilateral (*n* = 7) LLND. Resection of an upper recurrent nerve was performed on one patient. Prolonged intubation of 48.0 ± 16.9 h postoperatively was required in five patients. Tracheotomy was performed on one patient, who died 149 days after the surgical procedure due to complications of his tracheotomy. Histopathological analysis revealed papillary cancer (*n* = 12), follicular cancer (*n* = 2), medullary thyroid cancer (*n* = 3), and poorly differentiated cancer (*n* = 1). The mean duration of hospitalisation was 9.8 ± 5.8 days.

### 3.4. Compressive Hematoma

Ten patients (0.6%) presented with postoperative compressive hematoma (Table 1). No patients died. The initial surgical procedure consisted of total thyroidectomy (*n* = 10) without LND (*n* = 9) or was associated with ipsilateral CLNS (*n* = 1). In 6 out of 10 cases, surgical indication was a multinodular goiter. The time to onset of the hematoma was 6.8 ± 13.1 h. All patients were re-operated on and the origin of the bleeding was found in 8 out of 10 cases. The mean duration of hospitalisation was 5.9 ± 1.8 days.

### 3.5. Chyle Leakage

Thirteen (2.2%) patients had chyle leakage (Table 1). The initial intervention included ipsilateral CLND (*n* = 13), ipsilateral LLNS (*n* = 1), or ipsilateral (*n* = 10) or bilateral (*n* = 2) LLND. Fistula was diagnosed, on average, 1.6 ± 0.2 days after the procedure. In one patient, chyle leakage was identified during the intervention. The mean chyle flow observed in the drain on the first postoperative day was 175 ± 37.4 mL. Management began as soon as the diagnosis was suspected by a conservative treatment (compression dressing associated with a diet rich in medium-chain triglycerides). The mean duration of drainage was 8.1 ± 1.5 days. In one patient, conservative treatment failed despite the administration of somatostatin analogs from day 7 and thoracic canal ligation by thoracoscopy was performed at day 20 postoperatively. The mean hospital stay was 8.5 ± 1.9 days. No recurrence was observed.

### 3.6. Other Nerve Palsy

Ten patients (0.6%) had nerve palsy other than recurrent laryngeal nerve injury (Table 1). Lymph node dissection included unilateral CLND (*n* = 10) associated with ipsilateral (*n* = 8) or bilateral LLND (*n* = 2). Nerve damage was spinal nerve injury (*n* = 5) or Claude Bernard Horner’s syndrome (*n* = 5).

## 4. Discussion

In our study, we demonstrated that central lymph node dissection, and more particularly bilateral CLND, drastically increases the risk of transient and permanent postoperative hypoparathyroidism, while lateral lymph node dissection exposes a higher risk of recurrent laryngeal nerve palsy. Nowadays, the vast majority of patients with a diagnosis of thyroid cancer are submitted to total thyroidectomy. A high proportion also receive neck lymph node dissection even in the absence of documented lymph node metastases [16]. Noteworthily, there is little direct evidence of clinical benefit in terms of lower recurrence or mortality rates for thyroidectomy with prophylactic CLND over thyroidectomy alone, especially for the management of papillary thyroid carcinoma [3,4,5,6]. On the other hand, clear and consistent evidence demonstrates greater morbidity from neck lymph node dissection [7,8,9]. Thus, surgeons should balance the oncologic benefit of lymph node dissection with the risk of surgical complications. Among these, hypoparathyroidism remains the most common complication after thyroid surgery [17]. In our study, the rate of postoperative transient or permanent hypoparathyroidism was in the lower range of the incidence found in the literature, and consistent with a previous report from our department [13]. In a systematic review and meta-analysis of 115 studies, the median (IQR) incidence of temporary and long term post-surgical hypocalcaemia was 27% (19–38%) and 1% (0–3%), respectively [18]. Postoperative hypocalcaemia also represents a major cause of prolonged outpatient follow-up and hospital care cost [19]. In addition, the quality of life in patients with permanent hypoparathyroidism who require long-term calcium and vitamin D supplementation seems to be seriously affected [20]. The primary causes of postoperative hypoparathyroidism are direct injury, devascularisation, venous engorgement, or inadvertent excision of the parathyroid gland [21]. If the removal of one single parathyroid gland does not increase the risk of postoperative hypocalcaemia, the risk of hypocalcaemia is increased when fewer than three glands have been preserved in situ [13] and could lead to permanent hypoparathyroidism [22]. As demonstrated in a meta-analysis of over 3000 patients [23], we also observed in our cohort that transient hypocalcaemia was more common in patients undergoing ipsi or bilateral CLND in comparison with thyroidectomy alone. Moreover, multivariate analysis confirmed that CLND was an independent predictive factor of transient hypoparathyroidism. We also showed in the present multivariate analysis that bilateral CLND was associated with a (OR = 2.69, IC_95%_ [1.083–6.695], *p* = 0.033) higher risk of permanent hypocalcaemia (Table 4). Similar results also have been observed in a large series of 1087 patients submitted to thyroidectomy alone, or thyroidectomy plus ipsilateral or bilateral CLND, in which the authors demonstrated that bilateral CLND had a significantly higher rate of permanent hypoparathyroidism [24]. Recently, Salem et al. also demonstrated that CLND was a risk factor for permanent hypoparathyroidism (OR = 3.74, IC_95%_ [1.46–9.59]), based on the use of combined therapy 6 months after surgery [25]. Interestingly, they also found that node negativity was associated with a risk of permanent hypoparathyroidism (OR = 3.08, IC_95%_ [1.31–7.25]), suggesting that an extensive exploration of the central neck compartment might increase the risk of unintentional removal of the lower parathyroid glands. Our results also highlight the importance of in situ parathyroid gland preservation during total thyroidectomy, with or without LND, to avoid postoperative hypocalcaemia [26]. Here, we showed that the presence of parathyroid glands on the specimen is the main determinant of permanent postoperative hypoparathyroidism (OR = 4.42, IC_95%_ [1.727–11.291], *p* = 0.002) (Table 4). Thus, visual identification of all parathyroid glands and the complex vascular structures surrounding the parathyroid gland have been proposed to guarantee normal postoperative parathyroid function [27]. For that purpose, novel promising techniques using augmented and virtual reality in surgery have been developed to better identify parathyroid glands and their vascularization. Recently, Vidal Fortuny et al. demonstrated in a randomised clinical trial that intraoperative parathyroid gland angiography with indocyanine green (ICG) could predicate postoperative hypoparathyroidism and obviate the need for systematic blood tests and oral calcium supplementation [28]. In addition, a careful examination of the surgical specimen intraoperatively should also be performed to allow parathyroid auto-transplantation, if necessary, and to avoid inadvertent parathyroidectomy during thyroid surgery [29].

Likewise, RLN injury is a rare but potentially severe and life-threatening complication [30]. Symptoms can range from almost undetectable hoarseness in unilateral palsy to stridor and acute airway obstruction in bilateral damage [31]. Currently, RLN injury represents the main cause of medicolegal litigation after thyroid surgery [32]. Incidence of transient RLN injuries ranges from 1% to 30%, whereas permanent injuries are documented in 0.5% to 5% of patients, according to various studies and depending on the stringency of the postoperative laryngoscopy examination [33]. In our cohort, transient and permanent RLN injuries appeared in 8.9% and 2.8% of patients, respectively; well in line with previous published results [34]. Post-surgical vocal cord palsy is mainly caused by different intraoperative actions such as cutting, clamping, stretching, compressing, and heating [35]. Over the years, surgical strategy has advanced from non-visualization and avoidance of the RLN to the modern surgical technique of capsular dissection and direct visualization of the RLN to prevent neural damage [36]. As demonstrated by Misiolek et al., recurrent laryngeal nerves were not identified intraoperatively in 50% of patients with bilateral RLN injury after surgery [37]. The difficulty of dissecting and finding the RLN during cervical surgery also lies in the great anatomic variability of its position and sometimes is due to an early division in branches. Many methods have been recently described to help detect the RLN, such as the use of intraoperative neuromonitoring [38]. In our study, we focused on the impact of LND on postoperative RLN function. For that purpose, we chose to review an historical cohort before implementing intraoperative neuromonitoring to avoid bias due to the utilization of this novel technique. Thus, we found that transient or permanent RLN injuries were more frequent in patients who received ipsi or bilateral CLND, with or without LLND. However, multivariate analysis revealed that ipsi or bilateral CLND were not independent predictive factors of RLN injury. Interestingly, the study by Giordiano et al. showed that neither ipsi nor bilateral CLND increased the rate of transient or permanent hoarseness [24]. These results were also confirmed in a recent meta-analysis by Zhao et al., in which the risk of transient or permanent RLN injury was similar between thyroidectomy alone and thyroidectomy plus CLND [6]. Taken together, these results suggest that dissection of the recurrent laryngeal nerve during CLND does not provide further RLN injury than the dissection of the recurrent laryngeal nerve throughout thyroidectomy, and other surgical aspects should be taken apart. Lateral cervical neck dissection is commonly indicated in patients with proven lateral neck node involvement [2]. LLND, which normally entails levels IIa through Vb, exposes the patient to an increased risk of neural, vascular or lymphatic injuries. In the literature, a lack of evidence still remains concerning the impact of LLND on postoperative complications. Several retrospective studies showed increased complication rates in patients who had undergone LLND [39]. However, these results should be interpreted with caution. Indeed, most of these studies have compared the rate of complication between LND (i.e., CLND plus LLND) and no LND without making any distinction between central or lateral neck dissection [40,41,42,43,44,45,46]. Moreover, they do not, in their comparisons, precisely distinguish the different types of complications, surprisingly including hypocalcaemia, which is primarily due to CLND. In the present study, we obviously demonstrate that LLND and more especially bilateral LLND is a major contributor of post-surgical transient (OR = 10.434, IC_95%_ [4.943–22.026], *p* < 0.001), definitive (OR = 31.97, IC_95%_ [13.787–74.13], *p* < 0.001), and bilateral (OR = 6.512, IC_95%_ [1.695–25.021], *p* = 0.006) vocal cord palsy (Table 6). These results suggest that particular attention must be paid during the dissection of the vagus nerve to prevent surgical damage and to reduce vocal cord palsy. Furthermore, a surgical procedure that exposes both recurrent laryngeal nerve and both vagus nerves represents a major risk of bilateral RLN injury and should be performed by an experienced surgeon in a tertiary referral center.

## 5. Conclusions

Lymph node dissection during thyroidectomy in patients with differentiated carcinoma significantly increases postoperative morbidity. A wider knowledge of lymph-node-dissection-related complications associated with thyroid surgery could help surgeons to carefully evaluate the surgical and medical therapeutic options and to be able to give the patient adequate information about the benefits and the risks of surgery.

## Figures and Tables

**Table 1:** Clinical, surgical and histological characteristics in patients who underwent total thyroidectomy with or without lymph node dissection for thyroid cancer.

	No Lymph Node Dissection *n* = 1012	With Lymph Node Dissection *n* = 535	Total *n* = 1547	*p*
Patient characteristics				
Female sex	822 (81.2)	371 (69.3)	1193 (77.1)	<0.001
Age (yr)	50.9 ± 13.4	45.8 ± 16.0	49.1 ± 14.5	<0.001
Cervicotomy	12 (1.2)	24 (4.5)	36 (2.3)	<0.001
Cervical radiotherapy	5 (0.5)	2 (0.4)	7 (0.5)	0.738
Indication of thyroidectomy				
Benign goiter	848 (83.8)	43 (8)	891 (57.6)	<0.001
Toxic nodule or goiter	136 (13.4)	1 (0.2)	137 (8.9)	
Suspect thyroid nodule	28 (2.8)	491 (91.8)	519 (33.5)	
Surgery				
Thyroid weight (gr)	61.7 ± 52.4	37.3 ± 34.4	53.7 ± 48.6	<0.001
Number of parathyroid glands seen	3.2 ± 0.8	3.2 ± 0.8	3.2 ± 0.5	0.348
Number of parathyroid glands autotransplanted	0.1 ± 0.3	0.4 ± 0.7	0.2 ± 0.5	<0.001
Histology				
Micro-papillary thyroid cancer	688 (68)	92 (17.2)	780 (50.4)	<0.001
Papillary thyroid cancer	253 (25)	324 (60.6)	577 (37.3)	
Follicular thyroid cancer	62 (6.1)	36 (6.7)	98 (6.3)	
Medullary thyroid cancer	4 (0.4)	79 (14.8)	83 (5.4)	
Poorly differentiated cancer	3 (0.3)	3 (0.6)	6 (0.4)	
Tumor size (mm)	9.5 ± 14.0	21.3 ± 20.5	13.6 ± 17.4	<0.001
Stade T				
T1	863 (85.3)	282 (52.7)	1145 (74)	<0.001
T2	86 (8.5)	98 (18.3)	184 (11.9)	
T3–T4	62 (6.1)	155 (29)	217 (14)	
Stade N				
N0	0 (0)	136 (40)	136 (13.8)	<0.001
N1a	0 (0)	48 (9)	48 (3.1)	
N1b	0 (0)	166 (31)	166 (10.7)	
Parathyroid glands on specimen	40 (4)	76 (14.2)	116 (7.5)	<0.001

Data are presented as mean ± standard deviation or *n* (%). *p* values are derived from a Chi-squared test or Fisher’s exact test for nominal variables and from Student’s *t*-test for continuous variables.

**Table 2:** Surgical complications in patients who underwent total thyroidectomy with or without lymph node dissection for thyroid cancer.

	No Lymph Node Dissection *n* = 1012	With Lymph Node Dissection *n* = 535	Total *n* = 1547	*p*
Recurrent laryngeal nerve injury	68 (6.7)	131 (24.5)	199 (12.9)	<0.001
Transient	55 (5.4)	82 (15.3)	137 (8.9)	<0.001
Permanent	9 (0.9)	35 (6.5)	44 (2.8)	<0.001
Bilateral	4 (0.4)	14 (2.6)	18 (1.2)	<0.001
Hypoparathyroidism	128 (12.6)	107 (20.0)	235 (15.2)	<0.001
Transient	115 (11.4)	94 (17.6)	209 (13.5)	0.001
Permanent	13 (1.3)	13 (2.4)	26 (1.7)	0.096
Other surgical complications				
Hematoma	9 (0.9)	1 (0.2)	10 (0.6)	0.101
Chyle leakage	0 (0)	13 (2.4)	13 (0.8)	<0.001
Abscess	5 (0.5)	13 (2.4)	18 (1.2)	0.001
Other nerve injuries	0 (0)	12 (2.2)	12 (0.8)	<0.001

Data are presented as *n* (%). *p* values are derived from a Chi-squared test or Fisher’s exact test for nominal variables.

**Table 3:** Univariate analysis of predictive factors associated with transient and permanent hypoparathyroidism.

	No *n* = 1312	Transient *n* = 209	*p*	Permanent *n* = 26	*p*′
Patient characteristics					
Female gender	989 (75.4)	182 (87.1)	<0.001	22 (84.6)	0.360
Age (years)	49.8 ± 14.4	45.9 ± 14.4	<0.002	41.1 ± 13.6	0.002
Indication of thyroidectomy					
Benign goiter	791 (60.3)	91 (43.5)	<0.001	9 (34.6)	0.028
Toxic nodule or goiter	108 (8.2)	25 (12)		4 (15.4)	
Suspect thyroid nodule	413 (31.5)	93 (44.5)		13 (50)	
Surgery					
Thyroid weight (gr)	53.8 ± 48.6	53.4 ± 48.9	0.926	50.6 ± 47.1	0.753
Number of parathyroid glands seen	3.2 ± 0.8	3.3 ± 0.8	0.064	3.2 ± 0.8	0.971
Number of parathyroid glands autotransplantated	0.2 ± 0.4	0.4 ± 0.7	<0.001	0.3 ± 0.6	0.167
Lymph node dissection					
Central lymph node dissection					
Ipsilateral CLNS	33 (2.5)	5 (2.4)	1	1 (3.8)	0.491
Ipsilateral CLND	113 (8.6)	32 (15.3)	0.003	2 (7.7)	1
Bilateral CLND	137 (10.4)	48 (23.0)	<0.001	9 (34.6)	0.001
Lateral lymph node dissection					
Ipsilateral LLNS	54 (4.1)	9 (4.3)	0.852	1 (3.8)	1
Bilateral LLNS	101 (7.7)	20 (9.6)	0.337	3 (11.5)	0.448
Ipsilateral LLND	103 (7.9)	32 (15.3)	0.001	2 (7.7)	1
Bilateral LLND	40 (3)	12 (5.7)	0.062	5 (19.2)	0.001
Histology					
Micropapillary thyroid cancer	669 (51)	98 (46.9)	0.780	12 (46.2)	0.915
Papillary thyroid cancer	483 (36.8)	86 (41.1)		11 (42.3)	
Follicular thyroid cancer	85 (6.5)	12 (5.7)		1 (3.8)	
Medullary thyroid cancer	70 (5.3)	12 (5.7)		2 (7.7)	
Poorly differentiated cancer	5 (0.4)	1 (0.5)		0 (0)	
Tumor size (mm)	13.3 ± 17.2	15.4 ± 19.3	0.116	13.5 ± 14.4	0.960
Stade T					
T1	984 (75.1)	143 (68.4)	0.031	18 (69.2)	0.453
T2	155 (11.8)	27 (12.9)		2 (7.7)	
T3–T4	172 (13.1)	39 (18.7)		6 (23.1)	
Stade N					
N0	108 (8.2)	22 (10.5)	0.038	6 (23.1)	0.414
N1a	32 (2.4)	16 (7.7)		0 (0)	
N1b	118 (9.0)	43 (20.6)		5 (19.2)	
Parathyroid glands on specimen	79 (6.0)	28 (13.4)	<0.001	8 (30.8)	<0.001

Data are presented as mean ± standard deviation or *n* (%). *p* for comparison between no hypoparathyroidism and transient hypoparathyroidism. *p*′ for comparison between no hypoparathyroidism and permanent hypoparathyroidism. *p* and *p*′ values are derived from a Chi-squared test or Fisher’s exact test for nominal variables and from Student’s *t*-test for continuous variables.

**Table 4:** Multivariate analysis of predictive factors associated with transient and permanent hypoparathyroidism.

	Odds Ratio (CI 95%)	*p*
Transient hypoparathyroidism		
Female gender	2.48 (1.602– 3.843)	<0.001
Ipsilateral CLND	1.73 (1.07–2.786)	0.025
Bilateral CLND	2.09 (1.293–3.377)	0.003
Parathyroid glands on specimen	1.96 (1.467–2.617)	<0.001
Parathyroid glands autotransplantated	1.97 (1.207–3.216	0.007
Permanent hypoparathyroidism		
Age (years)	0.970 (0.944–0.997)	0.031
Bilateral CLND	2.692 (1.083–6.695)	0.033
Parathyroid glands on specimen	4.416 (1.727–11.291)	0.002

*p* values are derived from binary logistic regression model.

**Table 5:** Univariate analysis of predictive factors associated with transient, permanent of bilateral RLN injuries.

	No	Transient	*p*	Permanent	*p*′	Bilateral	*p*″
	*n* = 1348	*n* = 137		*n* = 44		*n* = 18	
Patient characteristics							
Female gender	1042 (77.3)	109 (79.6)	0.592	31 (70.5)	0.278	11 (61.1)	0.152
Age (years)	49.4 ± 14.2	45.6 ± 16.4	0.01	49.7 ± 14.4	0.876	54.2 ± 18.0	0.154
Indication of thyroidectomy							
Benign goiter	832 (61.7)	45 (32.8)	<0.001	9 (20.5)	<0.001	5 (27.8)	<0.001
Toxic nodule or goiter	122 (9.1)	14 (10.2)		1 (2.3)		0	
Suspect thyroid nodule	394 (29.2)	78 (56.9)		34 (77.3)		13 (72.3)	
Surgery							
Thyroid weight (gr)	54.1 ± 47.7	48.3 ± 44.9	0.189	48.4 ± 43.6	0.454	79.3 ± 113.8	0.374
Lymph node dissection							
Central lymph node dissection							
Ipsilateral CLNS	34 (2.5)	5 (3.6)	0.399	0	0.623	0	1
Ipsilateral CLND	107 (7.9)	29 (21.2)	<0.001	9 (20.5)	0.008	2 (11.1)	0.649
Bilateral CLND	127 (9.4)	34 (24.8)	<0.001	22 (50.0)	<0.001	11 (61.1)	<0.001
Lateral lymph node dissection							
Ipsilateral LLNS	52 (3.9)	7 (5.1)	0.487	4 (9.1)	0.097	1 (5.6)	0.512
Bilateral LLNS	102 (7.6)	19 (13.9)	0.020	2 (4.5)	0.728	1 (5.6)	1
Ipsilateral LLND	92 (6.8)	28 (20.4)	<0.001	14 (31.8)	<0.001	3 (16.7)	0.124
Bilateral LLND	24 (1.8)	13 (9.5)	<0.001	13 (29.5)	<0.001	7 (38.9)	<0.001
Histology							
Micropapillary thyroid cancer	715 (53)	53 (38.7)	<0.001	8 (18.2)	<0.001	3 (16.7)	<0.001
Papillary thyroid cancer	480 (35.6)	69 (50.4)		22 (50.0)		9 (50.0)	
Follicular thyroid cancer	90 (6.7)	4 (2.9)		2 (4.5)		2 (11.1)	
Medullary thyroid cancer	58 (4.3)	11 (8)		12 (27.3)		3 (16.7)	
Poorly differentiated cancer	5 (0.4)	0 (0)		0		1 (5.6)	
Tumor size (mm)	12.6 ± 15.3	15.5 ± 15.7	0.036	27.2 ± 26.6	0.001	37.4 ± 65.1	0.125
Stade T							
T1	1026 (76.2)	87 (63.5)	0.003	21 (47.7	<0.001	11 (61.1)	<0.001
T2	156 (11.6)	20 (14.6)		8 (18.2)		0	
T3–T4	165 (12.2)	30 (21.9)		15 (34.1)		7 (38.9)	
Stade N							
N0	107 (7.9)	16 (11.7)	0.018	9 (20.5)	0.028	4 (22.2)	0.614
N1a	32 (2.4)	12 (8.8)		2 (4.5)		2 (11.1)	
N1b	101 (7.5)	36 (26.3)		22 (50.0)		7 (38.9)	

Data are presented as mean ± standard deviation or *n* (%). *p* for comparison between no RLN injury and transient RLN injuries. *p*′ for comparison between no RLN injury and permanent RLN injuries. *p*″ for comparison between no RLN injury and bilateral RLN injuries. *p*, *p*′ and *p*″ values are derived from a Chi-squared test or Fisher’s exact test for nominal variables and from Student’s *t*-test for continuous variables.

**Table 6:** Multivariate analysis of predictive factors associated with transient, permanent or bilateral RLN injuries.

	Odd Ratio	*p*
Transient RLN injuries		
Ipsilateral CLND	2.207 (1.273–3.825)	0.005
Bilateral LLNS	2.738 (1.552–4.831)	0.001
Ipsilateral LLND	3.049 (1.711–5.434)	<0.001
Bilateral LLND	10.434 (4.943–22.026)	<0.001
Permanent RLN injuries		
Ipsilateral LLND	8.174 (3.794–17.612)	<0.001
Bilateral LLND	31.97 (13.787–74.13)	<0.001
Bilateral RLN injuries		
Bilateral CLND	5.566 (1.566–19.784)	0.008
Bilateral LLND	6.512 (1.695–25.021)	0.006
Tumor size	1.019 (0.997–1.041)	0.091

*p* values are derived from binary logistic regression model.

## Data Availability

The data are not publicly available due to the facility’s privacy policy.
